# Validity and Reliability of General Nutrition Knowledge Questionnaire for Adults in Uganda

**DOI:** 10.3390/nu9020172

**Published:** 2017-02-21

**Authors:** Richard Bukenya, Abhiya Ahmed, Jeanette M. Andrade, Diana S. Grigsby-Toussaint, John Muyonga, Juan E. Andrade

**Affiliations:** 1 Division of Nutritional Sciences, University of Illinois at Urbana-Champaign, Urbana, IL 61801, USA; bukenya2@illinois.edu (R.B.); dgrigs1@illinois.edu (D.S.G.-T.); 2 Department of Food Science and Human Nutrition, University of Illinois at Urbana-Champaign, Urbana, IL 61801, USA; ahmed36@illinois.edu; 3 School of Family and Consumer Sciences, Eastern Illinois University, Charleston, IL 61920, USA; jmevans@eiu.edu; 4 Department of Kinesiology and Community Health, University of Illinois at Urbana-Champaign, Urbana, IL 61801, USA; 5 Department of Food Technology and Human Nutrition, Makerere University, P.O. Box 7062, Kampala, Uganda; hmuyonga@yahoo.com

**Keywords:** nutrition knowledge questionnaire, validation, Uganda, adults

## Abstract

This study sought to develop and validate a general nutrition knowledge questionnaire (GNKQ) for Ugandan adults. The initial draft consisted of 133 items on five constructs associated with nutrition knowledge; expert recommendations (16 items), food groups (70 items), selecting food (10 items), nutrition and disease relationship (23 items), and food fortification in Uganda (14 items). The questionnaire validity was evaluated in three studies. For the content validity (study 1), a panel of five content matter nutrition experts reviewed the GNKQ draft before and after face validity. For the face validity (study 2), head teachers and health workers (*n* = 27) completed the questionnaire before attending one of three focus groups to review the clarity of the items. For the construct and test-rest reliability (study 3), head teachers (*n* = 40) from private and public primary schools and nutrition (*n* = 52) and engineering (*n* = 49) students from Makerere University took the questionnaire twice (two weeks apart). Experts agreed (content validity index, CVI > 0.9; reliability, Gwet’s AC1 > 0.85) that all constructs were relevant to evaluate nutrition knowledge. After the focus groups, 29 items were identified as unclear, requiring major (*n* = 5) and minor (*n* = 24) reviews. The final questionnaire had acceptable internal consistency (Cronbach α > 0.95), test-retest reliability (*r* = 0.89), and differentiated (*p* < 0.001) nutrition knowledge scores between nutrition (67 ± 5) and engineering (39 ± 11) students. Only the construct on nutrition recommendations was unreliable (Cronbach α = 0.51, test-retest *r* = 0.55), which requires further optimization. The final questionnaire included topics on food groups (41 items), selecting food (2 items), nutrition and disease relationship (14 items), and food fortification in Uganda (22 items) and had good content, construct, and test-retest reliability to evaluate nutrition knowledge among Ugandan adults.

## 1. Introduction

Malnutrition among school-aged children in Uganda remains high [1,2] as 22%–38% are considered stunted, 38% are anaemic, and 80% are iron-deficient, undermining national education efforts. This is partly due to poor feeding practices at homes and at schools [3]. Approximately 40% of the schools do not provide meals; 92% of rural children go to school without breakfast and 70% do not eat lunch at school. Additionally, few schools provide fruits, vegetables, animal source, and fortified food that can reduce micronutrient deficiencies [3]. Poor nutrition status manifests from the complex interaction of biological, intra- and interpersonal, and environmental factors that determine food choices and consumption among children [4]. At schools, head teachers’ actions are among the many interpersonal factors that can influence the school environment for children and, potentially, for their parents and other teachers. Head teachers are centrally positioned to promote nutrition in schools and their communities, often supporting nutrition interventions led by government and development partners [5,6]. Nonetheless, the ability of teachers and heads to influence healthy behaviours at schools might be limited by their knowledge of and attitudes towards nutrition [7].

The determinants of diet-related behaviours include biologically determined behavioural predispositions, experience with foods (physiological and social conditioning), intra- and interpersonal factors, and environmental factors [4]. Knowledge is a key determinant of behaviour, categorized within inter- and intrapersonal factors [4] and included within several theoretical frameworks such as the Social Ecological Model and the Social Cognitive Theory [8,9,10,11]. Increased nutrition knowledge has been associated with improved dietary habits and lower rates of obesity [12,13,14]. Furthermore, prior evidence indicates that gender differences in nutrition knowledge may explain differences in unhealthy behaviours, such overeating and smoking habits [15,16]. These studies suggest that monitoring and evaluating nutrition knowledge using valid instruments is an important component of health and nutrition education.

In Uganda, most studies have focused on evaluating knowledge, attitudes, and practices associated with nutrition in vulnerable populations, e.g., people with HIV-AIDS, infants, and young children [17,18]. These studies, however, have inadequately evaluated nutrition knowledge. The issue stems from two major factors; disagreement on a definition of nutrition knowledge and the use of instruments that are not validated [17,18]. Nutrition knowledge is defined as the understanding of basic facts about food and nutrition [19,20,21,22]. At a very basic level, nutrition knowledge should be defined by at least two of the following concepts during evaluation; food groups, balanced diets, current dietary guidelines, sources of nutrients, storage and preparation of food, use of food labels, and the relationship between nutrition and disease [19,20,21,22,23,24]. Most studies evaluating indicators of nutrition, including knowledge, in Uganda often adapt questionnaires from elsewhere, which do not undergo the complete validation process [17,18]. The lack of validated instruments negatively affects the quality of the resulting data and its extrapolation to a wider population, as well as limiting the ability to compare results from different studies [25]. Additionally, not validating survey tools reduces the ability of any given study to detect associations with other modulating factors, specifically feeding behaviors [25]. As a result, the use of low-quality data could negatively impact policies and programs and the effective use of resources. In Uganda, this was identified as a major gap to scaling up public nutrition action [26].

In order to support future nutrition education interventions in Uganda, this study sought to validate a general nutrition knowledge questionnaire (GNKQ) for adults using a systematic approach, involving college students and head teachers.

## 2. Materials and Methods

### 2.1. Review of Existing Nutrition Knowledge Questionnaires

A literature search in PubMed, Web of Science, and EBSCO using the keywords ‘nutrition’ AND ‘knowledge’ AND ‘validation’ between 1 January 1999 and 31 November 2014 revealed 154, 127, and 97 studies that evaluated nutrition knowledge, respectively. Studies were excluded if they did not focus on adults, were disease-focused questionnaires, were literature reviews, or did not provide validation of any type. Based on the review of titles and abstracts, validated questionnaires of nine studies were included (Table 1) [19,20,21,22,23,27,28,29,30,31,32]. These studies were mainly from Europe, North America, Australia, and Africa. The questionnaire from Parmenter and Wardle (1999) [19] was selected because of its: (a) widespread application to build similar questionnaires in several regions including Sub-Saharan Africa [20,31]; (b) reported validity and reliability; (c) inclusion of more than two domains of nutrition knowledge; and (d) target population.

Validation of the GNKQ followed steps described in a previous study [24] and procedures from other studies [20,21]. The selected questionnaire [19] had four constructs (110 items) evaluating nutrition knowledge: (i) expert recommendations (11); (ii) food groups (69); (iii) selecting food (10); and (iv) relationship between nutrition and disease (20). Food composition tables for Uganda [34] and Tanzania [35] were used to modify the GNKQ draft. Items on food fortification were adapted from a study in Uganda [36]. Currently, the Ugandan government and developing partners are promoting fortification [3,26]. Items on sources of nutrition information and demographic characteristics were included from another study [19,20].

### 2.2. Ethical Approval

The Institutional Review Board at the University of Illinois and the Uganda National Council for Science and Technology approved all research protocols. The Ministry of Health, District Education Offices, and Department Heads at Makerere University provided permissions to conduct studies. All subjects provided consent before participation.

### 2.3. Study 1: Assessment of Content Validity

#### 2.3.1. Subjects

An expert panel reviewed the content of the initial GNKQ draft. Contact information for the experts was obtained from the Nutrition Unit at the Ministry of Health, Kampala Uganda. Fifteen experts received the consent form along with the questionnaire electronically via Qualtrics Survey Software (2015; Qualtrics, Provo, UT, USA) [37]. Experts had over five years of work experience and knowledge of nutrition policies and programs in Uganda. Only five experts in the fields of health education (1), nutrition (2), agriculture (1), and education (1) completed the GNKQ. This number of experts was considered sufficient to review the instrument based on results from previous survey design studies, which used three to five experts to evaluate content validity [38,39].

#### 2.3.2. Procedures and Analyses

Experts reviewed and rated each item on its relevance, clarity, simplicity, and ambiguity to evaluate nutrition knowledge using a four-point Likert scale (1–4), representing low to high agreement. For content validity, the items under review included the question statement and its answer options. Expert reviews took at least two weeks to a month and were conducted twice, before and after face validation (Study 2). Scores on relevance were used to generate a content validity index, while clarity, simplicity, and ambiguity were used to pinpoint disagreement in the questionnaire’s structure. Also, experts recommended items to add/delete based on language, food, and nutrition policies in Uganda (Table 2). Scores evaluating the relevance of ‘items’ were dichotomized as explained in another study [40]. Levels ‘1’ and ‘2’ were assigned ‘0’, while ‘3’ and ‘4’ were assigned ‘1’. The content validity index (CVI) was the number of experts answering ‘3’ or ‘4’ (in agreement) divided by the total number of experts. The CVI was determined using Microsoft Excel 2010 (Microsoft, Redmond, WA, USA). The acceptable CVIs for items and constructs were 0.79 and 0.89, respectively [40]. Inter-rater reliability was determined using Gwet’s AC1. The command of ‘three raters or more’ in AgreeStat2013.3 (Advanced Analytics, LLC, Gaithersburg, MD, USA) [41] was used to estimate Gwet AC1. Benchmarks for Gwet’s AC1 were used, in which values <0.4 were poor, 0.4 to 0.75 were intermediate to good, and >0.75 were excellent [42].

### 2.4. Study 2: Assessment of Face Validity

#### 2.4.1 Subjects

Fifteen head teachers and twelve health workers from Kampala district filled out the modified GNKQ before attending one of three focus groups. Broad guidelines for conducting focus group discussions were used to determine the number and size of the groups [43]. The first two focus groups were comprised of seven (two female and five male) and five (two female and three male) head teachers. The third focus group was conducted with seven health workers (two female and five male), which included four nurses, two clinical officers, and a medical officer.

#### 2.4.2. Procedures and Analysis

Subjects reviewed and rated each item (statement and answer options) of the modified GNKQ based on clarity (Yes ‘1’ or No ‘2’) to the target group before attending a focus group. They provided reasons for unclear items. The CVI for items and constructs was calculated based on the number of participants in agreement (i.e., clarity = ‘yes’) divided by the total number of respondents [44]. The focus groups were conducted using the available recommendations [45]. Unclear items were reviewed during the focus groups. Each suggested modification was discussed until all participants agreed on clarity and there were no alternative opinions (saturation). Discussions during all focus groups were digitally recorded. Each focus group lasted two hours.

### 2.5. Study 3: Construct Validity and Test-Retest Reliability

#### 2.5.1. Subjects

Forty head teachers were recruited from private (52%) and government (48%) sponsored primary schools and from schools with (*n* = 23) and without (*n* = 17) a feeding program. The head teachers were adults (18–74 years) of both sexes (43% female). Additionally, second and third year undergraduate students from the nutrition (*n* = 52) and engineering (*n* = 49) departments at Makerere University participated in this study. The students were adults (18–34 years) of both sexes (48% female). Students from other years and departments were excluded.

#### 2.5.2. Procedures

The purpose of the pilot survey was to ascertain internal consistency, test-retest reliability, and construct validity of the items in the modified GNKQ after the second expert review. Students completed the questionnaire online via Qualtrics (2015). Head teachers completed the GNKQ at their schools. All subjects completed the same questionnaire after two weeks. The GNKQ consisted of the same five constructs and 139 items (Table 2), which represented the maximum score (i.e., 139 points). The answers were scored using a procedure previously reported [20], in which right answers were assigned one point and wrong ones no points. Other sections (i.e., sources of nutrition information and demographics) were not scored. All data were exported to the Statistical Package for Social Sciences (SPSS v23.0, IBM Corporation, Armonk, NY, USA) for analysis. The indices detailed below were calculated as described elsewhere [19].

#### 2.5.3. Analyses

Data from all subjects were used to ascertain item difficulty, item discrimination, internal consistency, and test-retest reliability but not for construct validity. Item difficulty was evaluated using benchmarks provided earlier [20]. Items are useful if they are answered correctly by 10%–90% of the respondents. Items that did not meet this criterion were removed from the analysis. Item discrimination is the ability for each item to discriminate between people with different levels of knowledge [19,46]. The item to total correlation coefficient, which is a correlation between an item score and the total score of the GNKQ, was obtained to evaluate item discrimination. Items with a coefficient <0.2 were removed from analysis. Internal consistency refers to the extent to which all items in the scale measure the same attribute. Cronbach alpha (α) was obtained from dichotomised (correct = 1, wrong = 0) values using SPSS23. Nutrition knowledge constructs and the whole GNKQ with α > 0.7 were considered with adequate internal consistency. Construct validity is the extent to which a test measures the attribute or variable it is intended to measure [19,47]. To ascertain construct validity, two populations with assumed different knowledge are commonly used [24]. Thus, differences in the GNKQ’s scores between nutrition vs. engineering students were used to assess construct validity. Differences in nutrition knowledge were evaluated using the Mann-Whitney *U* Test. Test-retest reliability demonstrates that the results produced are consistent over time. Head teachers’ and students’ scores over the two-week period were used in this estimation. Since the data were not normally distributed, the Spearman Rank correlation coefficient (r) was used. The acceptable test-retest reliability was *r* ≥ 0.7.

## 3. Results

The GNKQ for Uganda is available in supplementary materials.

### 3.1. Study 1: Content Validation

The overall CVI on the relevance of items to evaluate nutrition knowledge after the first and second round was 0.89 and 0.97, respectively (Table 3). After the first round of expert review, only items on the constructs of ‘nutrition and disease relationship’ had an acceptable content validity index (CVI > 0.9). The expert agreement reliability on the relevance of the contents improved (Gwet’s AC1 from 0.71 to 0.96) after face validity on the second expert review.

### 3.2. Study 2: Face Validation

A total of 29 items from all the constructs were considered unclear (CVI < 1) by the head teachers and health workers. Only five of the 29 items had major changes as reviewed during the focus groups. The participants agreed that other items were clearly understood even though they were unsure of the definite answers. The items that were modified in the focus groups are found in the supplementary materials.

### 3.3. Study 3: Internal Consistency, Test-Retest Reliability and Construct Validity

Characteristics of the participants are presented in Table 4. The results of internal consistency and test-retest reliability are presented in Table 5. For all participants, the internal consistency after the first and second round of surveys and before deleting any items were; knowledge of ‘food groups’ (α = 0.81 and 0.81), ‘relationships between nutrition and diseases’ (α = 0.77 and 0.84), ‘food fortification’ (α = 0.94 and 0.93), ‘expert recommendations’ (α = 0.46 and 0.56), and ‘selecting food’ (α = 0.40 and 0.38), respectively.

After the deletion of items using the criteria of item difficulty and discrimination, ‘selecting food’ for the first and second round was α = 0.92 and α = 0.84. However, the entire ‘expert recommendations’ construct was eliminated during analysis because of unacceptable internal consistency (α = 0.59). The test-retest reliability for items in the constructs on ‘expert recommendations’ and ‘selecting food’ were unacceptable (*r* < 0.7) before deleting the items with unacceptable item difficulty, discrimination, and internal consistency. Apart from ‘expert recommendations,’ other constructs had acceptable (*r* > 0.7) test-retest reliability after deleting items. Differences in scores for the whole survey and each construct after the second round of data collection showed that nutrition students had higher scores (*U* = 29, *p* < 0.001) than their counterparts in engineering (Table 6). Head teachers total scores were 43.9 ± 9.7.

## 4. Discussion

Currently, there is no valid tool to collect general nutrition knowledge in Uganda or the great majority of countries in Sub-Saharan Africa. The initial GNKQ (133 items) was reviewed to include commonly consumed food items in Uganda [34,35], the concept of ‘food fortification,’ and current nutrition-related guidelines and policies in Uganda (Table 2) [26,48,49]. Five experts in nutrition-related disciplines reviewed the first GNKQ drafts resulting in high consensus on the relevance of constructs (CVI and Gwet’s AC1 > 0.96). Previous studies have used at least three experts to review similar questionnaires aimed at evaluating the nutrition knowledge of adults [19,20,27,28,29,30,31]. None of these studies, however, reported the level of agreement among experts on the relevance of the contents of the different constructs of the GNKQs. The content validity index (CVI) is a one proportion agreement method that has been used in the past to quantitatively estimate content validity [40,44]. Experts in survey evaluation suggest reporting at least two measures of agreement [40,50,51,52]. Relying on only CVI is not adequate because it might inflate agreement among experts since there is no adjustment for chance agreement [50]. Gwet’s AC1 was used as a second measure of agreement because of the small sample size of experts and stability concerns of the Kappa statistic. Gwet’s AC1 is a more stable measure of interrater agreement reliability than the commonly used kappa statistic [42]. The recommended minimum item-CVI is 0.8, while the scale-CVI is 0.9, when using a panel of five or more experts. In the case that less than five experts review the questionnaire, there should be a perfect agreement, i.e., a CVI of 1.0 [40,44]. A Gwet’s AC1 above 0.4 represents intermediate to excellent agreement reliability [42]. Therefore, the GNKQ for Uganda had adequate content validity.

The results from overall internal consistency, test-retest reliability, and construct validity of the questionnaire before and after the deletion of items based on item difficulty and discrimination were adequate and comparable to other studies [19,27,28,32,46]. Results from the validation of a similar questionnaire for adults (*n* = 125 college students) in Turkey [27] yielded poor internal consistency for knowledge on ‘dietary recommendations’ (α = 0.47) and ‘choosing foods’ (α = 0.43). These results are comparable to those in this study after the first round of validation with students. Similar to previous studies [19,27,28,32,46], the internal consistency (α > 0.7) and test-retest reliability (*r* > 0.7) were adequate for three domains of nutrition knowledge i.e., sources of nutrients, food selection, and diet and disease relationship. The poor results on ‘expert recommendations’ may be due to discordant interpretations of nutrition messages partly attributed to limited nutrition education promotion and a lack of unified dietary guidelines in Uganda [24]. Low internal consistency has also been attributed to the heterogeneity of populations with a varied education background [22,34]. The current study included teachers and students with different education backgrounds. Administering the GNKQ to a larger homogenous sample (e.g., head teachers only) could improve these findings on internal consistency. All versions of the GNKQ validated in different countries showed good construct validity [19,27,28,32,46]. Similar to these studies, nutrition students scored higher than engineering students on the overall score and in each topic, demonstrating that the GNKQ has adequate construct validity.

Even though results obtained in this study support the validity of the questionnaire to evaluate the nutrition knowledge of adults, there are some limitations. In this study, the focus was on developing a nutrition knowledge questionnaire for adults with the ultimate goal to evaluate nutrition knowledge among head teachers, as they are often recruited in the implementation of government nutrition policies. The educational attainment of head teachers is higher than most of the low-income population in Uganda, which requires further adaptation of the GNKQ for populations with limited education. In addition, the GNKQ mainly evaluates declarative rather than procedural knowledge. The questionnaire, however, could be used as a first step in the evaluation of attitudes and behaviors toward nutrition. Although very common in the literature, the use of students to evaluate construct validity may not be appropriate to establish this attribute in a more diverse adult population.

The development of instruments to collect valid and reliable data is critical for both scientists and practitioners, particularly in Sub-Saharan Africa. The items in the revised GNKQ draft had good validity and reliability in a sample obtained from Kampala district. Kampala is represented by diverse population groups including urban, peri-urban, rural, agricultural, cultural groups, the affluent, and the poor. This diversity potentiates the ability of the GNKQ to obtain valid results when used in other regions. Potentially, the questionnaire can be used to collect information on nutrition knowledge and its change after interventions among various population groups, especially opinion leaders or influential agents such as teachers, agriculture extension agents, and health workers. Moreover, the adaptability of the questionnaire can be evaluated in other countries in Sub-Saharan Africa. 

## 5. Conclusions

The results demonstrate that the final draft of the GNKQ has items that can be used to collect valid and reliable nutrition knowledge data from adults in Uganda. The items had acceptable construct, content and face validity, internal consistency, and test-retest reliability. The topic on ‘expert opinions’ requires further validation. Future studies will continue the validation of this questionnaire in a larger population as well as address its predictive validity on nutrition and health behavior changes among adults.

## Figures and Tables

**Table 1:** Studies selected in the final review to draft the initial general nutrition knowledge questionnaire (GNKQ) for adults in Uganda.

Region/Country	Ref.	Year	Target Group	No. of Topics on Nutrition	Were Validation Steps Reported during the Psychometric Tool Development? (Yes/No)				
					Content Validity	Face Validity	Internal Consistency	Construct Validity	Test-Retest Reliability
Uganda	[17]	2010	Adults	4	No	No	No	No	No
	[18]	2005	Adults	1	No	No	No	No	No
	[33]	2015	Adults	1	No	No	No	No	No
Sub-Saharan Africa	[20]	2005	Youth	5	Yes	Yes	Yes	Yes	No
	[31]	2008	Adults	1	Yes	Yes	Yes	Yes	No
Americas	[23]	1997	Adults	2	Yes	No	Yes	Yes	No
	[29]	2003	Adults	2	Yes	Yes	Yes	Yes	Yes
	[32]	2009	Adults	6	Yes	Yes	Yes	No	No
Europe	[19]	1999	Adults	4	Yes	No	Yes	Yes	Yes
	[27]	2012	Adults	4	Yes	Yes	Yes	Yes	Yes
	[30]	2013	Adults	2	Yes	Yes	Yes	Yes	Yes
Australia	[28]	2008	Adults	4	Yes	Yes	Yes	Yes	Yes

**Table 2:** Units of analysis for content validity and reliability analyses.

Topic on General Nutrition	Study 1		Study 3	
	First Draft	After Content and Face Validity	After Content and Face Validity	After difficulty, Discrimination, & Internal Consistency Analysis
	Items (Statements/Answer Options)		Items (Answer Options)	
Expert Recommendations	4/16	4/16	16	0
Food groups	21/70	17/66	66	41
Selecting foods	10/10	10/10	10	2
Relationship of nutrition and disease	10/23	11/24	24	14
Food fortification	3/14	5/23	23	22
Total	48/133	47/139	139	79

**Table 3:** Content validity index (CVI) and reliability of expert agreements before and after face validation.

Topics (No. of Items after 1st/2nd Review)	First Round			Second Round		
	CVI	Gwet’s AC1	*p*-Value	CVI	Gwet’s AC1	*p*-Value
Expert Recommendations (4/4)	0.85	0.60	<0.05	0.90	0.89	<0.05
Food groups (21/17)	0.88	0.81	<0.05	0.93	0.92	<0.05
Food choices (10, 10)	0.84	0.62	<0.05	1.00	1.00	<0.05
Relationship of nutrition and disease (10/11)	0.96	0.91	<0.05	1.00	1.00	<0.05
Food fortification (3/5)	0.73	0.23	>0.05	0.92	0.91	<0.05
*Whole Questionnaire (48/47)*	0.89	0.71	<0.05	0.97	0.96	<0.05

**Table 4:** Characteristics of the participants in the test-retest study.

Characteristic	Nutrition Students (*n* = 40)		Engineering Students (*n* = 37)		Head Teachers (*n* = 40)	
	*n*	%	*n*	%	*n*	%
*Gender*						
Male	14	35	26	70.3	23	57
Female	26	65	11	29.7	17	43
*Age*						
18–24	35	87	33	89		
25–34	5	13	4	11	8	20
35–44					10	25
45–54					15	38
55–64					6	15
65–74					1	2
≥75						
*Education*						
Ordinary Secondary school					2	5
High School (A’ level)					1	2
Technical college					1	2
Diploma					17	43
Degree					14	35
Post graduate degree					7	13
*Number of children*						
None	37	92	36	97	2	5
1	2	5	1	3	2	5
2	1	3			8	20
3					5	13
4					9	22
≥5					14	35

**Table 5:** Internal consistency and test-retest reliability of the items in the GNKQ before and after the deletion of items based on item difficulty and discrimination.

Topic on General Nutrition	Internal Reliability (α)				Test-Retest Reliability	
	Before		After		Before	After
	*Round 1*	*Round 2*	*Round 1*	*Round 2*		
Expert recommendations	0.46	0.56			0.55	
Food groups	0.81	0.81	0.87	0.85	0.80	0.80
Selecting foods	0.40	0.38	0.92	0.85	0.57	0.77
Relationship of nutrition and disease	0.77	0.84	0.89	0.91	0.78	0.84
Food fortification	0.94	0.93	0.94	0.94	0.79	0.80
Total	0.93	0.94	0.95	0.95	0.88	0.89

**Table 6:** Nutrition knowledge scores of nutrition and engineering students.

Topic (Max Score)	Nutrition (*n* = 40)			Engineering (*n* = 37)			Mean Diff.	Mann-Whitney U	*p*-Value
	Min	Max	Mean (SD)	Min	Max	Mean (SD)			
Food groups (41)	28.0	41.0	36.4 (3.0)	16.0	38.0	27.6 (5.6)	8.8	127.0	<0.001
Selecting foods (2)	0.0	2.0	1.1 (1.0)	0.0	2.0	0.6 (0.9)	0.5	560	0.036
Relationship of nutrition and disease (14)	10.0	14.0	13.0 (1.1)	0.0	11.0	5.4 (2.6)	7.6	42.5	<0.001
Food fortification (22)	8.0	22.0	16.5 (3.7)	0.0	19.0	4.9 (6.1)	11.6	80.0	<0.001
Total (79)	56.0	76.0	67.0 (4.9)	20.0	67.0	38.7 (11)	28.3	29.0	<0.001

Min: minimum value; Max: maximum value; SD: standard deviation, *n*: sample size, Mean Diff.: mean difference.

## Supplementary Materials

The following are available online at http://www.mdpi.com/2072-6643/9/2/172/s1.
