# Supplementary material for: Validity and Reliability of General Nutrition Knowledge Questionnaire for Adults in Uganda
Source: Nutrients. 2017 Feb 21;9(2):172. doi: 10.3390/nu9020172 (PMC5331603; doi:10.3390/nu9020172)
Supplement: Supplementary file 1 [file nutrients-09-00172-s001.pdf]

Nutrition Survey for Adults

Nutrition survey
This is a survey, not a test. Your answers will help identify which dietary advice to adult people that is not clear.
1. It is important that you complete the questionnaire by yourself.
2. Your answers will remain anonymous.
3. If you do not know the answer, mark “not sure” rather than guessing.

A. The first few items are about what advice you think experts are giving in the community.

1. Do you think health experts recommend that people should be: i) eating more of the under listed food, ii) the same amount of the under listed food, iii less of the under listed food? (tick one box per food)

	More	Same	Less	Not sure
Vegetables	☐	☐	☐	☐
Sugary foods	☐	☐	☐	☐
Meat	☐	☐	☐	☐
Starchy foods	☐	☐	☐	☐
Fatty foods	☐	☐	☐	☐
High fibre or roughage	☐	☐	☐	☐
Fruits	☐	☐	☐	☐
Salty foods	☐	☐	☐	☐

2. How many servings of fruit and vegetables a day do you think health/ nutrition experts are advising people to eat? (*One serving could be, for example, an apple, an orange or a handful of dodo, carrots or bugga*)

_____ Servings of Fruits ☐ Not sure

_____ Servings of vegetables ☐ Not sure

3. Which food fats do health/ nutrition experts say should be reduced? (tick one box per food)

	Yes	No	Not sure
Pork	☐	☐	☐
Fish	☐	☐	☐
Margarine/Butter	☐	☐	☐
Beef	☐	☐	☐
Ground nuts	☐	☐	☐

4. What version of dairy food products do health/ nutrition experts say people should eat? (tick one)

Full cream milk ☐

Yoghurt ☐

Should not take any dairy foods ☐

Not sure ☐

B. Experts classify foods into groups. We are interested to see whether people are aware of what foods are in these groups.

1. Do you think the underlisted foods are high or low in added sugar? (tick one box per food)

	High	Low	Not sure
Bananas	☐	☐	☐
Maize meal (posho)	☐	☐	☐
Soda	☐	☐	☐
Plantain (matooke)	☐	☐	☐
Quencher juice	☐	☐	☐
Ripe Mangoes	☐	☐	☐

2. Do you think the under listed foods are high or low in fat? (tick one box per food)

	High	Low	Not sure
Chapatti	☐	☐	☐
Margarine e.g. blueband	☐	☐	☐
Beans	☐	☐	☐
Sausages	☐	☐	☐
Honey	☐	☐	☐
Eggs	☐	☐	☐
Ground nuts	☐	☐	☐
Bread	☐	☐	☐
Simsim	☐	☐	☐
Ghee	☐	☐	☐

3. Which of the foods below do you think experts classify under starchy foods group? (tick one box per food)

	Yes	No	Not sure
Milk	☐	☐	☐
Rice	☐	☐	☐
beans	☐	☐	☐
fish	☐	☐	☐
sweet potatoes	☐	☐	☐
Maize porridge	☐	☐	☐

4. Do you think the listed foods are high or low in salt? (tick one box per food)

	High	Low	Not sure
Sausages	☐	☐	☐
Chapatti	☐	☐	☐
Smoked fish	☐	☐	☐
Beef	☐	☐	☐
Amaranths (dodo)	☐	☐	☐
Groundnut paste	☐	☐	☐

5. Do you think the under listed foods are high or low in protein? (tick one box per food)

	High	Low	Not sure
Chicken	☐	☐	☐
Mangoes	☐	☐	☐
Beans	☐	☐	☐
Beef	☐	☐	☐
Stiff porridge (posho)	☐	☐	☐
Yogurt	☐	☐	☐

6. Do you think the under listed foods are high or low in fibre/roughage?(tick one box per food)

	High	Low	Not sure
Stiff porridge (posho)	☐	☐	☐
Bananas	☐	☐	☐
Eggs	☐	☐	☐
Beef	☐	☐	☐
Cabbage	☐	☐	☐
Ground nuts	☐	☐	☐
Fish	☐	☐	☐
Irish potatoes with skins	☐	☐	☐
Chicken	☐	☐	☐
Beans	☐	☐	☐

7. Do you think health/ nutrition experts call the listed foods healthy alternative to beef? (tick one box per food)

	Yes	No	Not sure
Sweet potatoes	☐	☐	☐
Sausage	☐	☐	☐
Beans	☐	☐	☐
Ground nuts	☐	☐	☐
Yogurt	☐	☐	☐
Bread	☐	☐	☐

8. A glass of unsweetened fruit juice is a good alternative to the real fruit. (tick one)

   Agree ☐ Disagree ☐ Not sure ☐

9. Brown sugar is a healthy alternative to white sugar. (tick one)

   Agree ☐ Disagree ☐ Not sure ☐

10. There is more protein in a glass of whole milk than in a glass of yoghurt. (tick one)

    Agree ☐ Disagree ☐ Not sure ☐

11. Liquid cooking oil (e.g. mukwano) contains less fat than solid oil (e.g. kimbo). (tick one)

    Agree ☐ Disagree ☐ Not sure ☐

12. Which of these bread types contain the most vitamins and minerals? (tick one)

White ☐ Brown ☐ Wholegrain ☐ Not sure ☐

13. Which of the two foods do you think contain high energy: butter or regular margarine (e.g. blue band)? (tick one)

Butter ☐ Margarine (e.g. Blue band) ☐ Both the same ☐ Not sure ☐

14. There is more calcium in a glass of whole milk than a glass of yoghurt. (tick one)

Agree ☐ Disagree ☐ Not sure ☐

15. Which one of the following food groups provides the highest energy for the same weight? (tick one)

Sugar ☐ Starchy foods ☐ Fibre/ roughage ☐ Fat ☐ Not sure ☐

16. Table sugar has a lot of vitamins and minerals.

True ☐ False ☐ Not sure ☐

17. Which of these foods are high or low in vitamin A?

	Low	High	Not sure
Carrots	☐	☐	☐
Spinach	☐	☐	☐
Yellow fleshed sweet potatoes	☐	☐	☐
Maize	☐	☐	☐
Matooke	☐	☐	☐
Rice	☐	☐	☐

C. The next few items are about choosing foods.

Please answer what is being asked and not whether you like or dislike the food!
For example, suppose you were asked . . . .
If a person wanted to cut down on fat, which meat would be best to eat?'
a) Chicken
b) Beef
c) Beef sausage
d) Pork
If you didn't like chicken, but knew it was the right answer, you would still tick chicken.

1. Which would be the best choice for a low fat, high fibre/ roughage snack? (tick one)

   Roasted groundnuts ☐
   Ripe mango ☐
   Sim sim ☐
   Not sure ☐

2. Which would be the best choice for a low fat, high fibre meal? (tick one)

   Beef and plantain (matooke) ☐
   Groundnuts and plantain (matooke) ☐
   Beans and Plantain (matooke) ☐
   Fish and plantain (matooke) ☐
   Not sure ☐

3. Many people eat thick porridge (posho) with beans. Which do you think is healthier? (tick one)

   Thick porridge (posho) with fried beans and dodo ☐
   Thick porridge (posho) with fried beans and potato chips ☐
   Not sure ☐

4. If a person wanted to reduce the amount of fat in their diet, which would be the best choice? (tick one)

   Fish, grilled ☐
   Sausages, grilled ☐
   Beef steak, grilled ☐
   Pork chop, grilled ☐
   Not sure ☐

5. If a person wanted to reduce the amount of fat in their diet, but didn't want to give up chips, which one would be the best choice? (tick one)

   Thick cut chips ☐

   Thin cut chips ☐

   Not sure ☐

6. If someone felt like eating something sweet, and at the same time trying to cut down on sugar, which would be the best choice? (tick one)

   Honey on bread ☐

   Flavoured yoghurt ☐

   Plain digestive biscuit ☐

   Banana with plain yoghurt ☐

   Not sure ☐

7. Which of these snacks would be the best choice as a lower fat option? (tick one)

   Cassava fresh, fried ☐

   Chapati fried ☐

   Samosa beans filling, fried ☐

   Not sure ☐

8. If a person wanted to reduce the amount of salt in their diet, which would be the best choice? (tick one)

   Roasted pork with pineapple ☐

   Mushroom and ground nuts ☐

   Vegetables with soy sauce ☐

   Sausages with cabbages ☐

   Not sure ☐

9. Which consistence of porridge do you think should be given to the children? (tick one)

   Thick porridge ☐

   Watery porridge ☐

   Not sure ☐

10. Why do think we should give the type of porridge in question 9? (tick one)

    It is less thick and nutrients can easily be absorbed ☐

    There are more ingredients and nutrients can be easily absorbed ☐

    I am not sure ☐

D. This section is about health problems or diseases.

1. I think that one should protect themselves from diseases by not eating various kinds of foods.

   (Tick one)

   True ☐ False ☐ Not sure ☐

2. How can you recognize that children are eating less food? (tick all that apply)

   Children do not play ☐
   Frequent infections ☐
   Physical weight loss ☐
   Not sure ☐

3. I think that children without appetite should be forced to eat. (tick one)

   True ☐ False ☐ Not sure ☐

4. Are you aware of any major health problems or diseases that are related to a low intake of fruit and vegetables? (tick one)

   Yes ☐ No ☐ Not sure ☐

   If yes, what diseases or health problems do you think are related to a low intake of fruit and vegetables? (*Name at least one*)

5. Are you aware of any major health problems or diseases that are related to a low intake of fibre or roughage?

   Yes ☐ No ☐ Not sure ☐

   If yes, what diseases or health problems do you think are related to fiber or roughage? (*Name at least one*)

6. Are you aware of any major health problems or diseases that are related to how much sugar people eat?

   Yes ☐ No ☐ Not sure ☐

   If yes, what diseases or health problems do you think are related to eating sugary foods like sweets? (*Name at least one*)

7. Are you aware of any major health problems or diseases that are related to how much salt or sodium people eat?

   Yes ☐ No ☐ Not sure ☐

   If yes, what diseases or health problems do you think are related to salt? (*Name at least one*)

8. Are you aware of any major health problems or diseases that are related to the amount of fat people eat?

Yes ☐ No ☐ Not sure ☐

If yes, what diseases or health problems do you think are related to fat? (*Name at least one*)

9. Do you think the underlisted foods help to reduce the chances of getting certain kinds of cancer? (tick one box per food)

	Yes	No	Not sure
Eating more fibre or roughage	☐	☐	☐
Eating less sugar	☐	☐	☐
Eating less fat	☐	☐	☐
Eating less salt	☐	☐	☐
Eating more fruits and vegetables	☐	☐	☐
Eating less preservatives/ additives	☐	☐	☐

10. Do you think the underlisted foods will help prevent heart disease? (tick one box per food)

	Yes	No	Not sure
Eating more fibre or roughage	☐	☐	☐
Eating less fat	☐	☐	☐
Eating less salt	☐	☐	☐
Eating more fruits and vegetables	☐	☐	☐
Eating less preservatives/ additives	☐	☐	☐

11. Have you heard of antioxidant vitamins?

Yes ☐ No ☐ If No, continue with question 13

12. Which one of these is more likely to raise people's blood cholesterol level? (tick one)

- Vegetables ☐
- Fruits ☐
- Animal fats ☐
- Plant oils ☐
- Legumes ☐
- Not sure ☐

13. Have you heard of antioxidant vitamins?

Yes ☐ No ☐ If No, continue with Section E

14. If you responded YES to question 12, do you think these are antioxidant vitamins? (tick one box per vitamin)

	Yes	No	Not sure
Vitamin A	☐	☐	☐
B complex vitamins	☐	☐	☐
Vitamin C	☐	☐	☐
Vitamin E	☐	☐	☐
Vitamin K	☐	☐	☐

E. Experts recommend consuming foods with more vitamins and minerals. Food companies <u>add</u> them through a process called <u>fortification</u> (i.e. <u>fortified foods</u>).

1. Have you heard about, seen or used any fortified food on the market?

   Yes ☐ No ☐ ….If No is your answer proceed to Section F

2. Which of the following foods have nutrients added (fortified) in Uganda? (tick one box per food)

	Yes	No	Not sure
Vegetable oil	☐	☐	☐
Sugar	☐	☐	☐
Maize flour	☐	☐	☐
Wheat flour (engano)	☐	☐	☐
Currypowder	☐	☐	☐
Salt	☐	☐	☐
Millet flour	☐	☐	☐
Soda	☐	☐	☐

3. Which of these foods has iodine mandatory added. (tick one box per food)

	Yes	No	Not sure
Bread	☐	☐	☐
Vegetable oil	☐	☐	☐
Powdered milk	☐	☐	☐
Table salt	☐	☐	☐
Wheat flour (engano)	☐	☐	☐

4. Which of these foods has vitamin A mandatory added? (tick one box per food)

	Yes	No	Not sure
Bread	☐	☐	☐
Vegetable oil	☐	☐	☐
Powdered milk	☐	☐	☐
Table salt	☐	☐	☐
Wheat flour (engano)	☐	☐	☐

5. Have you seen any of these logos on the label of packages of foods with added nutrients (fortified). (tick one box per logo)

	Yes	No	Not sure
	☐	☐	☐
	☐	☐	☐
	☐	☐	☐
	☐	☐	☐

F. Sources of Health and Nutrition Information: In this section we are seeking for the sources of information you use.

1. Where do you get your information about nutrition? (tick that apply)

Source	Yes	No
Schools	☐	☐
Peers/friends	☐	☐
Health personnel	☐	☐
Parents/Guardian	☐	☐
Radio/TV/ magazines/books	☐	☐
Internet	☐	☐
Other (specify)…………………………………………………	☐	☐
………………………………………………………..………………	☐	☐

2. From the choices you have selected above, how would you rate them as sources of information? (circle one choice)

1= Very unreliable; 2= Unreliable; 3= Reliable; 4= Very reliable

Source	Very unreliable	Unreliable	Reliable	Very reliable
Schools	1	2	3	4
Peers/friends	1	2	3	4
Health personnel	1	2	3	4
Radio/TV/ magazines/books	1	2	3	4
Parents/ guardians	1	2	3	4
Internet	1	2	3	4
Other (specify)………………… …………………………………..	1	2	3	4

G. Food safety: Expert recommend that we handle food with more safety to avoid contamination. Which of these statements do you think are true of false

1. I think cooked meat/fish/chicken sold on the streets may not be safe to eat because it may be undercooked. (tick one)

   True ☐ False ☐ Not sure ☐

2. I think cooked meat sold on streets may not be safe to eat because it is kept long before cooking. (tick one)

   True ☐ False ☐ Not sure ☐

3. I think it is important to clean dirty utensils with only water to avoid food contamination. (tick one)

   True ☐ False ☐ Not sure ☐

4. Food leftovers should be kept in a cool place because higher temperatures make germs grow faster. (tick one)

   True ☐ False ☐ Not sure ☐

5. I think raw fish, meat and ofals should be put together with other cooked food during storage. (tick one)

   True ☐ False ☐ Not sure ☐

6. What do you think are the key moments that one should wash hands to prevent germs from reaching food?. (Name at least two moments)

   ____

   ____

H. Finally, we would like to ask you a few questions about yourself

1. What is your gender?
   - Male ☐
   - Female ☐

2. How old are you?
   - Less than 18 ☐
   - 18 -24 ☐
   - 25 -34 ☐
   - 35 - 44 ☐
   - 45 -54 ☐
   - 55 - 64 ☐
   - 65 – 74 ☐
   - More than 74 ☐

3. Indicate the number of children you have(Tick one)
   - None ☐
   - 1 ☐
   - 2 ☐
   - 3 ☐
   - 4 ☐
   - More than 4 ☐

4. Do you have any children under 18 years, living with you?
   - Yes ☐
   - No ☐

5. What is the highest level of education you have completed?
   - Primary school ☐
   - Secondary school ☐
   - O level ☐
   - A level ☐
   - Technical or tertiary certificate ☐
   - Diploma ☐
   - Degree ☐
   - Post-graduate degree ☐

6. Do you have any nutrition related qualification?

   Yes ☐

   Please specify

   No ☐

7. Are your currently a nutrition student?

   Yes ☐

   Please specify

   No ☐

8. If you have a partner, does he/she have any nutrition related qualification or student?

   Yes ☐

   Please specify

   No ☐

9. If you have a partner, what is his/her job? If he/she is not working now, what is his/her usual job? (please be specific):

10. Are you on a special diet?

   Yes ☐

   Please specify:

   No ☐

THE END

Thank you very much for your time. If there are any comments you would like to make about this questionnaire, please do so below, they would be very welcome.
